# Challenging Behaviors in Children with Nonverbal Autism: A Questionnaire to Guide the Design of a Wearable Device for Biomarker Recording

**DOI:** 10.3390/s25072009

**Published:** 2025-03-23

**Authors:** Anne-Sophie Weber, Camilla Barbini, Olivia Vidal, Laura M. Ferrari, Dimitri Thellier, Alexandre Derreumaux, Esma Ismailova, Florence Askenazy, Susanne Thümmler

**Affiliations:** 1 Université Côte d’Azur, CoBTek, 06100 Nice, France; 2 University Department of Child and Adolescent Psychiatry, Children’s Hospitals of Nice, CHU-Lenval, 06200 Nice, France; 3 Université Côte d’Azur, Inria Sophia Antipolis, Stars, 06902 Sophia-Antipolis, France; 4 Istituto di Biorobotica, Scuola Superiore Sant’Anna, 56127 Pisa, Italy; 5 Centre Hospitalier Esquirol, Département d’Information Médicale, 87000 Limoges, France; 6 Mines Saint-Etienne, CMP, BEL, 13541 Gardanne, France

**Keywords:** non or minimally verbal ASD, severe language impairment, behavioral disorder, stress, wearable device, electrophysiological biosignals

## Abstract

**Highlights:**

Approximately 25% of individuals affected with ASD exhibit severe language impairments and are more likely to have challenging behavioral issues. Recent research focuses on early detection and prevention of crises by utilizing biomarkers measuring stress signals before the onset of the crisis. Regarding design requirements, the trunk appears to be the preferred location for wearable devices in this population. The arm and wrist can be an alternative with encouraging results. These locations offer interesting prospects for integrating devices into clothing or connected bracelets. The utilization of wires should be discouraged in the design of wearable devices for this specific population. Acceptance could be improved through prior desensitization.

**What are the main findings?**

- A total of 67.5% of respondents support a wearable stress detection device for nmvASD children, preferably integrated into clothing, placed on the trunk, and be wireless.

**What is the implication of the main finding?**

- These findings guide the development of tailored tools to improve behavioral management in this population.

**Abstract:**

Children with non- or minimally verbal autism (nmvASD) commonly display sensory and emotional dysregulations leading to extremely stressful situations that trigger challenging behaviors which are often difficult to treat. Nonetheless, this population remains rarely studied in clinical research. Recent methods use electrophysiological biomarkers as diagnostic tools to detect stress signals, which may be useful in anticipating situations or conditions leading to challenging behaviors in nmvASD. A specific questionnaire was created in order to identify the characteristics of nmvASD children and gather the opinions of future users (parents and caregivers) on the design of a wearable device able to collect stress-related electrophysiological data. The results indicate that approximately 67.5% of respondents (*n* = 40) would be interested in such a device, both in outpatient and inpatient settings. In 70% of cases, prolonged contact with an object on the trunk is always well accepted by the child. This location was also preferentially chosen by 57.5% of respondents for such a wearable device. The presence of wires could be problematic in 82.5% of cases. About 65% of respondents find it far better to integrate these wearable devices directly into the clothing. These results will help in the development of devices specifically developed for the nmvASD population to enhance their care for behavioral disorders and based on user-center design.

## 1. Introduction

Autism Spectrum Disorder (ASD) is a neurodevelopmental disorder that manifests early in childhood. It is characterized by both persistent deficits in communication and social interactions within various contexts, as well as restricted and repetitive patterns of behaviors, interests, or activities [1].

Currently, it is estimated that ASD affects approximately 1% of the global population, with a male-to-female ratio of 4:1. Its prevalence has been reported to increase in recent years [2,3]. However, some authors call for caution and attribute this trend to the improvement of diagnostic methods or a better detection of the disability by relatives [4,5]. ASD etiology appears to be multifactorial, featuring a genetic predominance over environmental factors [6]. About 25% of individuals affected with ASD exhibit severe language impairments, with or without the presence of intellectual deficit [7].

### 1.1. Sensory Particularities

Individuals with ASD often exhibit specific sensory perceptions that differ from the general population. This complex process involves hypo- and hypersensitivities impairing various sensory systems such as the auditory, proprioceptive, and tactile systems, leading to challenges in integrating and interpreting sensory stimuli, disrupting emotional modulation and expression [8,9,10].

As the self-regulation of these systems has been altered, this results in heightened emotional stress states leading to maladaptive behavioral responses.

While behavioral disorders are prevalent in the ASD population [11,12], they are more pronounced in children with severe ASD and those with associated language disorders [11,13,14,15].

### 1.2. Behavioral Issues

The inability to communicate is in fact an aggravating factor, leading to frustration and anger as a response to an accumulation of various stressful events. The severity of those behavioral disorders varies and may include challenging behaviors such as self-harm, explosive anger outbursts, or acts of aggression towards family members or caregivers [16,17,18,19,20].

These challenging behaviors are predictive factors for the level of impairment and the primary cause for institutionalization and therefore play a major role in public health issues [21]. Moreover, behavioral disorders can also substantially impact the mental health of caregivers and relatives, resulting in major care difficulties and causing additional distress for those ASD patients [22].

Up to now, several methods exist to reduce behavioral disorders, such as psychotherapy, communication trainings [23], cognitive behavioral therapies [24], PECS (Picture Exchange Communication System), EIBI (Early Intensive Behavioral Intervention), ABA (Applied Behavior Analysis) methods [25], and environmental adaptation [26]. However, these methods do not always provide satisfactory results [27].

Pharmacological methods, on the other hand, are only used in the most severe cases. They usually are not fully effective, causing short and long-term side effects and therefore result in an unsatisfactory benefit–risk ratio [28,29].

Hence, several studies emphasize the need to prevent behavioral issues early on, rather than addressing them during a crisis [16,30].

### 1.3. Stress Biomarkers

Recently, new methods have emerged exploiting electrophysiological biomarkers to evaluate stress levels and predict behavioral disorders [16,31]. When one experience emotional stress, several physiological changes can indeed be observed. Those changes are measurable, reliable, and mostly involuntary. Such signals that can be monitored include heart rate, electrodermal activity (EDA) [32,33,34] or galvanic skin response (GSR) [35], body temperature [36,37] and respiratory rate. Additionally, heart rate (HR) measurements, coupled with respiratory rate (RSP), are also recognized as reliable tools for identifying stress markers [38,39]. Also, heart rate variability (HRV), combined with GSR and RSP measurements, is widely regarded as one of the best biomarkers measurable with wearable devices [40] and is among the most commonly used in stress research [38].

These biomarkers prove to be more reliable than analyzing behavioral responses such as facial expression, speech, gesture, or posture [37,41,42] and the possibility of the use of cameras will be very limited outside standardized research and hospital settings, such as in-home care and care institutions for those children.

In ASD children, HR, HRV, and EDA (GSR) have been described as baseline and stress condition physiological parameters [43,44,45,46,47]. Also, the regulation of the autonomic nervous system appears to be impaired compared to controls with lower para- and higher sympathetic regulation [48,49,50] and might thus be used as a specific marker of anxiety-related arousal in this population [51].

Therefore, electrophysiological biomarkers might allow better anticipation of an impending behavioral crisis by early personalized interventions [27,52].

### 1.4. Wearable Devices for Children with ASD

However, devices used to collect electrophysiological data are usually poorly suited for children with severe, non or minimally verbal ASD, which can result in the loss of information or provoke additional stress to the child [53,54,55]. Moreover, in the current literature, few studies are related to such children [56] which, as described earlier, exhibit more behavioral problems resulting in a significant impairment in quality of life [12,13,14,15,21].

To the best of our knowledge, only one not yet replicated study in a small population (n = 20) concentrates on nmvASD children [52]. Based on EDA and cardiovascular activity recorded by a wristband wearable device, aggressive behaviors could be predicted one minute ahead.

Although there is a strong interest in the modern literature regarding the use of connected and robotic devices to support the detection and management of stress in children from neurotypical populations [57] little is known about stress in non-verbal ASD children [58].

Although recent studies explored the design of such tools for the pediatric populations [59], no design considerations have yet considered the perspectives of end users regarding usage specifically adapted to severe ASD [60].

For all these reasons, we have chosen to study the specificities of the nmvASD population in order to define which factors need to be considered for the elaboration of a future device able to detect electrophysiological stress biomarkers preceding challenging behaviors. Thus, our study’s first step for use-centered design [61] of a stress detection device in this specific population frequently presenting challenging behaviors of high impact for daily life.

In this study, we thus aim to explore our hypothesis—by means of a user-centered questionnaire—that a wearable real-time stress detection device might be beneficial for both patients and caregivers. We also intend to investigate the extent to which such a device could be accepted by participants, taking into account specific factors such as sensitivity to physical contact and the ability to recognize and communicate emotions. Finally, we seek to determine which body area(s) would be most suitable for wearing such a device.

Our results provide key insights into the most suitable device design, placement, and integration strategies, ensuring that future technologies are tailored to the specific sensory and behavioral needs of nmvASD children. They might be used to guide engineers and researchers in technological sciences for the design of such a device in this specific population.

## 2. Materials and Methods

The study has been systematically proposed to all families assessed at the Autism Resource Center in Nice, as well as to caregivers specializing in the care of this population, such as psychiatrists, psychologists, psychomotor therapists, and specialized educators. Initial contact was made either at the time of evaluation or via telephone. Reminders were sent to participants to increase the response rate.

The questionnaire was presented as an initiative aimed at gathering feedback concerning a specific child on a technological device in order to measure physiological parameters in nmvASD children during stress or internal agitation which might be related to challenging behaviors and thus used for anticipation and prevention.

This study was conducted over a 24-month period between February 2021 and February 2023.

The inclusion criteria were as follows (Appendix A): (1) Being either a parent, a guardian, or a healthcare professional (2) of a child of 3 to 18 years who has been diagnosed by a pediatric psychiatry ASD expert using standardized assessments with (3) ASD associated with (4) severe language impairment (being either non-verbal or minimally verbal). There have been no exclusion criteria for this study.

Non or minimally verbal autism has been considered as a limited use of vocabulary, typically with fewer than 20 words as proposed by the literature [56]. Due to the severe language impairments of the study population, the questionnaires were filled out by the child’s parent, caregiver, or healthcare professional.

A specific questionnaire was developed with neuropsychologists and doctors of the Autism Resources Center of Nice, clinical researchers of CoBTek laboratory and engineers of Ecoles des Mines and Inria (Table 1).

It is composed of five sections administered in about 15 min. The first two parts collect (1.) general data, as well as (2.) sensitivity characteristics and acceptability of skin contacts of variable intensities on different body locations (wrist, arm, trunk, head, and forehead) as well as past experiences the nmvASD child went through. The proposed locations have been selected to be as less intrusive as possible, with low friction, and low bending. The third part dealt with (3.) the identification of emotions. The fourth section explored (4.) the potential utility and tolerability of a device made of both an ultra-lightweight and non-invasive temporary tattoo—able to record the physiological signals—and a thicker than the tattoo but relatively flexible electronic component to collect and send the data. The last section of the questionnaire collected (5.) opinions regarding various technical parameters to ensure optimal acceptability by the nmvASD child.

The LimeSurvey online platform has been used through the University Côte d’Azur to generate and send the questionnaires, as well as to collect data, which were then extracted in Microsoft Excel format.

Cronbach’s alpha was used to calculate the internal consistency of the questionnaire and showed acceptable to excellent internal consistency (emotion section 0.76, utility section 0.90, and sensory sections 0.97).

The qualitative variables were described by their distribution in frequencies and proportions. A Shapiro–Wilk test was conducted to confirm that the population did not follow a normal distribution. A 95% confidence interval was calculated for each proportion, and a Pearson’s chi-square test (or Fisher’s Exact test if applicable) was conducted against the null hypothesis that the proportions of binary variables are equivalent to 50%. Statistical analysis thus explored the factors which might influence the acceptability of wearing a device, such as tolerance to different types of contact on various body areas, device placement, and the requirements for the design, such as color or the presence of wires. The comparative analysis concerned the parent vs. caregiver group, as well as children with severe vs. mild to moderate ASD. This article adheres to the principles of the STROBE guidelines.

The data were processed using Jamovi software version 2.3.26.0. The acceptable threshold for the *p*-value was set at 0.05.

All procedures have been approved by the Local Ethical Committee (Comité d’Ethique de l’Université Côte d’Azur, CERNI, accepted 14 Novembre 2020, reference n° 2020-75).

## 3. Results

This section presents the findings from the study, covering participant demographics, sensory sensitivity, emotional understanding, and preferences for wearable devices. A total of 40 questionnaires were completed by parents and professionals, providing insights into the characteristics and daily challenges of children with severe autism. The results highlight key considerations regarding sensory tolerance, behavioral patterns, and the feasibility of using wearable technology to support these children.

### 3.1. Population

Out of the 60 individuals contacted, 14 declined to take part in the study, and 6 did not follow up. In total, 40 questionnaires were completed, 15 of which (37.5%) were filled out by parents and 25 (62.5%) by professionals (caregivers or specialized educators).

The studied children’s characteristics are described in Table 2 and Table 3 and the average age was 11.5 years (SD = 4.59), ranging from 4.17 years to 17.92 years.

Among the 40 referred children, a majority (65%) suffered from severe autism. In the sample, most children exhibited at least one behavioral issue per day, and 45% of them had more than 15 episodes per day, according to the response to the “Frequency of challenging behavior” question in the questionnaire.

### 3.2. Sensitivity

The results regarding the acceptance of different types of contact based on location are presented in Table 3. The only significant difference found between children with severe autism and others relates to the level of pressure touch, generally less tolerated by children with severe autism, regardless of location (chest pressure: *p* = 0.002, arm *p* < 0.001, forehead: *p* = 0.018). From the sample, 20 children (50%) had already had an ECG, 16 had EEG, 15 had temporary tattoos, and 13 had a bracelet or watch. The acceptance rates were 20%, 18.8%, 33.3%, and 30.8%, respectively.

**Table 3:** Variation in contact acceptance by location.

Type of Contact	Location	Always	Often	Sometimes	Rarely	Not at all	N/A *
Light brief touch (like a stroke)	Trunk	**50%**	20%	20%	7.5%	0%	2.5%
	Arm	27.5%	**35%**	27.5%	7.5%	0%	2.5%
	Wrist	**42.5%**	27.5%	20%	7.5%	2.5%	0%
	Head	**37.5%**	22.5%	27.5%	10%	2.5%	0%
	Forehead	**37.5%**	30%	17.5%	7.5%	5%	2.5%
Light sustained touch (like giving a hug)	Trunk	25%	30%	32.5%	7.5%	2.5%	2.5%
	Arm	22.5%	15%	20%	22.5%	10%	10%
	Wrist	32.5%	25%	27.5%	10%	0%	5%
	Head	32.5%	15%	35%	10%	5%	2.5%
	Forehead	25%	25%	25%	10%	5%	10%
Pressure touch (like putting a plaster)	Trunk	17.5%	15%	15%	25%	7.5%	20%
	Arm	22.5%	15%	20%	22.5%	10%	10%
	Wrist	20%	15%	20%	15%	17.5%	12.5%
	Head	17.5%	5%	25%	17.5%	22.5%	12.5%
	Forehead	10%	12.5%	20%	25%	17.5%	15%
Light sustained touch with an object (like clothing friction)	Trunk	**70%**	20%	2.5%	2.5%	5%	0%
	Arm	60%	22.5%	10%	2.5%	2.5%	2.5%
	Wrist	**57.5%**	20%	10%	2.5%	2.5%	2.5%
	Head	**37.5%**	17.5%	15%	22.5%	22.5%	0%
	Forehead	**42.5%**	20%	5%	17.5%	17.5%	2.5%
Moving contact (like applying a cream)	Trunk	30%	27.5%	20%	10%	5%	7.5%
	Arm	**37.5%**	25%	15%	10%	5%	7.5%
	Wrist	27.5%	30%	20%	10%	5%	7.5%
	Head	22.5%	20%	22.5%	17.5%	10%	7.5%
	Forehead	25%	22.5%	17.5%	20%	7.5%	7.5
Contact with a liquid substance (water)	Trunk	**80%**	7.5%	7.5%	2.5%	0%	2.5%
	Arm	**77.5%**	15%	7.5%	0%	0%	0%
	Wrist	**80%**	12.5%	7.5%	0%	0%	0%
	Head	**57.5%**	17.5%	7.5%	12.5%	5%	0%
	Forehead	**62.5%**	17.5%	10%	7.5%	2.5%	0%

* N/A: Not Available/Unknown.

### 3.3. Emotions

Table 4 features the results regarding the comprehension of the studied children’s emotions and behaviors by the questionnaire respondents. In general, many respondents present difficulties in identifying them. In terms of detecting inappropriate behaviors, although 42.5% can frequently or always anticipate them, 55% can only do so occasionally or never. This is true with no significant difference between parents and professionals.

### 3.4. Wereable Device

**Figure 1:**
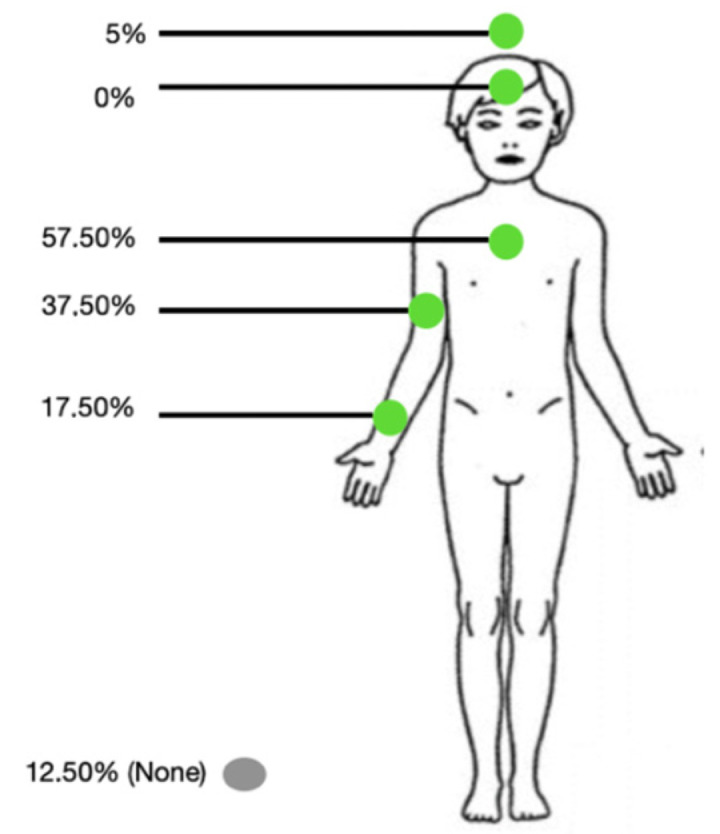
Preferred body location for the device.

A device to prevent the child’s crises was deemed useful by the majority (67.5%). About the tolerance of a cutaneous device, seven individuals (17.5%) believe the sensors, as described in the questionnaire, might not be tolerated by their child. The majority (57.5%) think that sensors at trunk level (chest/back) would be more tolerated by the child compared to other locations (see Figure 1).

### 3.5. Technical Parameters

For the presence of wires, a large majority (82.5%) believe they would be bothersome. The results indicate that for 11 children (27.5%), there could be a risk of inappropriate or even dangerous behavior associated with their presence.

A significant majority of respondents (75.5%) felt the presented sensors would be of noticeable (62.5%) or significant (15%) help in managing their child’s behavioral issues. Sixty-five percent of respondents think it would be very interesting if integrated into clothing.

Concerning its color, the two following options appear to be the most appropriate: skin color (57.7%) or transparent (52.5%).

In general, there are no significant differences between the parents and caregivers.

## 4. Discussion

This study aims to guide the design of wearable real-time stress detection devices for nmvASD children by evaluating possible benefits and acceptance using a specific questionnaire for both patients and caregivers of those children, considering sensitivity to physical contact, emotional recognition, and the optimal body area for device placement. Out of 60 contacted individuals, 40 completed the questionnaire, with 37.5% responses from parents and 62.5% from professionals. The children studied had an average age of 11.5 years, with 65% diagnosed with severe autism. Most children experienced daily behavioral issues, with 45% having more than 15 episodes per day. The results showed significant differences in tolerance to pressure touch, with children with severe autism being less tolerant. Various types of contact and devices have been evaluated, with temporary tattoos and bracelets showing higher acceptance rates. Many respondents struggled with identifying children’s emotions and predicting inappropriate behaviors, with 55% unable to anticipate these behaviors consistently. A majority (67.5%) found the concept of a wearable device useful for the prevention of crises. Concerns were noted regarding the tolerance of sensors and the presence of wires, with 82.5% finding wires potentially bothersome and 27.5% identifying risks of inappropriate behavior due to wires. The trunk (chest/back) was considered the most tolerable placement (57.5%). The preferred device colors were skin color (57.7%) or transparent (52.5%). A significant majority (75.5%) believed the sensors would help manage behavioral issues, and 65% expressed interest in integrating the device into clothing. The study supports the hypothesis that a wearable stress detection device could be beneficial for nmvASD children, with considerations for design and placement critical for acceptance.

The need for improving care for nmvASD children is substantial. Nevertheless, there are, to date, few effective methods to act early on these symptoms, which significantly impact on their quality of life and may lead to long-term comorbidities. However, in recent years, there has been a growing interest in wearable devices, which prove to be a promising sector to predict challenging behaviors, while also contributing to the research and allowing a better understanding of this condition.

Several projects are in the preliminary testing phase or not yet validated, but most of them are focused on projects targeting the general population and are therefore poorly suited for individuals with nmvASD and for real-time detection and everyday use [53,55,62].

The specificity and diversity of clinical presentations of the nmvASD population pose a challenge for the device’s designers and require prerequisites that will be essential for its future efficiency.

Our study results also allow to refine the selection of biomarkers to be used for the ASD population. EEG, a reliable marker of stress due to its correlation with stress levels and its high temporal resolution [40,63] is emerging as a promising biomarker in the literature. However, according to our findings, EEG devices do not appear suitable for the nmvSD population due to the location and somewhat invasive nature of such equipment. Other biomarkers that can be measured using less invasive methods, such as EDR, HR, and HRV as well as respiratory signals (RSP) should be prioritized. Some of the latter electrophysiological biomarker might be recorded using common wrist-based wearable devices but might be challenging due to frequent movement artifacts in this population, especially of extremities.

To the best of our knowledge, this study is the first to assess the characteristics of the specific population of nmvASD children before designing such a device, aimed at improving their daily lives.

Another study, involving 31 teenagers and adults exhibiting ASD without intellectual deficiency suggested a prototype in the form of a glove with multiple sensors on the fingers [64]. Using a questionnaire, various parameters were analyzed, including comfort, flexibility, safety, and weight. It is noteworthy that this study also found a similar genuine interest from caregivers in the presented device. A majority of parents (72%) expressed a strong interest in monitoring the physiological signals of their children and therefore, better understanding their anxiety levels and emotions. However, this study did not take into account the presence of language disorders nor their severity. Moreover, the device’s preferred location has not been investigated, and we have strong reservations about the suitability of wearing a glove for nmvASD individuals due to the intensity of sensory issues they may experience.

Our study suggests that a wearable solution for stress detection may prove useful both for in- and out-patients and domestic settings, as anticipating behavior disorder episodes can be challenging both for caregivers and parents. Therefore, considering the input of end users appears to be crucial before designing such products, particularly in order to adapt them to this population’s specific needs. Thanks to this questionnaire, we were able to define the difficulties and specific characteristics these nmvASD children face, as well as their level of tolerance towards various stimuli in their daily lives.

Our results suggest the most suitable location for prolonged use of a wearable cutaneous device would be the trunk (70% of cases, always tolerated). It is at this level that children would indeed accept the most varied sensory stimulations, and it is also the preferred location chosen by the relatives and caregivers of children under their care. The arm and wrist also seem to be favorable and suitable locations, with a total acceptance of 60% and 57.50%, respectively.

The trunk (chest or back) has the advantage of being an ideal area for recording various electrophysiological signals (heart and respiratory rates, temperature, perspiration, etc.) that may be harder to measure at the extremities for some. Integrating such sensors into clothing also appears to be better suited compared to more peripheral locations.

The arm and wrist would have the advantage of easily capturing stereotypies [52], which could be relevant for predicting behavioral crises. Unlike the trunk, which provides stable conditions for recording physiological biomarkers such as respiratory rate and electrodermal activity, the arm is more directly involved in motor behaviors, including repetitive movements that are commonly observed in this population.

In addition, in recent years, other devices have been developed specifically for the pediatric population. One device which might also be of interest for stress-related biomarker recording in nmvASD children is a clock-like wristband recording several physiological parameters (https://kiddo.health/, accessed on 5 March 2025). Its use might be limited in nmvASD children presenting sensory discomfort and aversion to wearing accessories in this specific region but might be suitable for the group of nmvASD children accepting the device. Also, a study that involved children with ASD, 85% of whom were minimally verbal, was able to collect data via a connected wristband, and the device seemed to be well accepted by the children after they followed a desensitization protocol [52]. Therefore, this solution should not be excluded, and prior desensitization seems to have a significant positive impact on the tolerance of any devices and should, in our opinion, be systematically carried out.

However, we noted that stimulation on the forehead or head is less tolerated than others, regardless of the type of stimulation. For example, prolonged touch by an object is never tolerated in 17.50% and 22.50% of cases, respectively, and similar results were found for pressure touch.

In addition, responses suggest that a device with wires would not be very well tolerated. This is probably one of the reasons why ECGs were poorly accepted in this population (in 55% of cases, never accepted). It seems essential for us that future designers take these limitations into account.

The prototype presented in our questionnaire was based on devices in the current literature, featuring significant improvements in thickness in the nano/micrometric scale, and increased flexibility [65,66,67].

However, due to design constraints and the electronic part, the device would actually be thicker and more visible than initially described, generating a more significant sensory stimulation and potentially causing noticeable discomfort for the child. There is still progress to be made regarding the thickness and visibility of the electronic component if it were too visible, there would be a risk of it being torn off or generating friction with clothing, for example.

The placement of the fastening and the safety of the material appear to be crucial factors to consider before designing the product. It is not uncommon with behavioral disorders to involve self-harming and this must also be taken into account in the manufacturing process. The must-have criteria to take into consideration when designing such a device would be a safe and difficult to access a product that is flexible and without wires [68].

The results from the questionnaire highlighted a significant interest in integrating such a device into clothing like a T-shirt. This alternative could be promising and has already been the subject of an experimental study [69] on individuals with ASD exhibiting behavioral issues. The device appeared to have been well accepted [70]. In addition, wearable textile humidity sensors have demonstrated high detection performance, reproducibility, stability, and flexibility [71] and thus might also be suitable for stress detection in nmvASD. Integrating it directly into clothing would favor better acceptance, as it would be less visible and easily incorporated into an everyday object. However, to date, no study has been performed to assess the acceptance of such a device on a larger-scale sample of nmvASD children. This could lead the way for future investigations to be carried out about such a device.

Certain materials using conductive polymers can allow the recording of physiological parameters thanks to sensors directly embedded in textiles [72] and an ECG signal might be successfully recorded, even when the patient is in motion [73].

All these advancements are promising and fall in line with our results and could help draft a simplified manufacturing process for various biomedical devices on textiles. Miniaturizing of electronic devices as well as the development of monitoring of patients in various ecological settings like home would therefore also benefit the nmvASD population [74,75].

A significant limitation of current detection systems of anxiety is their inability to differentiate physiological arousal caused by anxiety from that triggered by other states, such as physical activity. However, an algorithm [76] addresses this issue by minimizing false detections related to user motion and accurately identifying arousal states even during periods of movement. This contributes to the growing body of evidence supporting the potential of wearable technologies for detecting and managing anxiety in real-world environments.

Moreover, the incorporation of artificial intelligence as a joint analysis of electrophysiological biomarkers related to stress has been reported [76]. This approach would permit a multimodal analysis of several physiological markers such as ECG, electrodermal activity, and respiratory rate [32,33,34,35]. Data collection could be performed across multiple dimensions, each responding in their own way to stress factors. Analyzing these data on a global combined level could be essential to obtain even more accurate results. This method facilitates identifying and understanding better responses to stress, and could potentially serve as a valuable tool in preventing the onset of related disorders. In addition, the simultaneous analysis of several biomarkers would allow taking into account the individual electrophysiological profiles of ASD children responding to stress and related to the occurrence of challenging behaviors.

Lastly, ensuring the quality of recorded signals is a critical aspect of designing an effective wearable device for children with nmvASD. Several technical variables must be considered. The device must be capable of continuously capturing physiological signals over extended periods while maintaining high accuracy, which necessitates an optimized sampling rate to balance resolution and power consumption. Additionally, the form factor plays a crucial role in acceptability; the device should be lightweight, flexible, and non-intrusive to minimize sensory discomfort. Regarding data processing, logging data for offline analysis can be beneficial for long-term monitoring, whereas real-time streaming may be necessary for immediate intervention, such as detecting acute stress responses. Future studies should focus on optimizing these parameters to enhance the practicality and usability of such devices in real-life settings.

This study, to the best of our knowledge, is the first to measure, in the population of children and adolescents with nmvASD, the acceptance of a wearable cutaneous device, assessed both among parents and caregivers. It could help evaluate the viability of such devices for future studies.

### Limitations

The main limitation of our study is the absence of a direct evaluation of the acceptability of the biofeedback device corresponding to the results we present. Unfortunately, this will be the focus of our future research, the aim of the present study is to help guide the development of devices for the specific population of nmvASD children. Also, as biomarker research is rare and very challenging in children with severe autism, preliminary steps such as our study might help to reverse this trend.

Another limitation might be the number of participants and the absence of more detailed patient data beyond those presented in Table 2. In fact, correlation with detailed patient characteristics will be essential during the next steps of development, in particular concerning individual sensory specificities. In addition, further research needs to continue to include end users such as parents and caregivers and should be shared with patient associations.

A minor limitation is the potential of reporting bias as we mitigated by cross-referencing respondents’ statements with objective data from the medical records of the children being followed.

## 5. Conclusions

According to our findings, the trunk appears to be the preferred location for wearable devices in children with ASD and severe language disorders. The arm and wrist can also be considered as an alternative, with encouraging results. These locations offer interesting prospects such as connected wristbands or clothing-integrated devices. Regardless of the nature of the devices, it seems essential to emphasize they should be as discreet as possible, with minimal electronic components and no associated wires. Their acceptance could also be improved through prior desensitization carried out by the child’s attendant and caregiver.

## Figures and Tables

**Table 1:** Structure of the study questionnaire.

Section	Theme	Question	Question Style
1. General Information	Relationship with child	N/A	Multiple choice
	Child Characteristics	Age Sex Severity of disorder Level of verbal communication Frequency of challenging behavior	Open-ended Binary Multiple choice Binary Multiple choice
2. Sensitivity	Assessment of acceptance of various contacts based on each location	Light, brief touch Light, sustained touch Pressure touch Light, sustained contact with object Moving contact Contact with a liquid substance	Likert scales *
	Previous experience and acceptation	Bracelet, watch Connected watch ECG, EEG Temporary tattoo Wires	Binary and Likert scales *
3. Emotions	Capacity of recognition	Happiness Pain Intensity of pain Sickness Stress/anxiety Cause of anxiety	Likert scales *
	Characteristics Challenging behavior Communication of emotions	Hypo/hypersensitivity Capacity to anticipate Used a technologic/non technologic method and utility	Binary and Likert scales *
4. Device presentation	Picture and information about the device	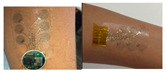	N/A
	Potential utility and tolerability according to the respondent	Utility in general Acceptation and tolerability Utility at home/hospital/caring unit Utility all day	Likert scales *
5. Technical parameters	Advice for conception	Location Wires Sensors Colors T-shirt integration Suggestions	Multiple choice Likert scales * Likert scales * Multiple choice Likert scales * Open comment

* 5-item Likert Scale.

**Table 2:** Sample characteristics.

Study Population		*N* = 40	% of Total
Gender	Male	29	72.5%
	Female	11	27.5%
ASD Severity	Mild	2	5%
	Moderate	9	22.5%
	Severe	26	65%
	N/A *	3	7.5%
Verbal Communication Skills **	Nonverbal	20	50%
	Minimal verbal	20	50%
Challenging Behaviors ***	Never or rarely	7	17.5%
	1 time or more each day	15	37.5%
	15 times or more each day	11	27.5%
	30 times or more each day	7	17.5%

* N/A: Not Available/Unknown. ** Nonverbal: uses less than five functional words; Minimal verbal: uses less than 20 functional words. *** Challenging behaviors: Hetero-aggressive behavior, self-injury, meltdowns, repetitive movements, inappropriate response to the environment.

**Table 4:** Emotion and Behavior Understanding.

Item	Always	Often	Sometimes	Rarely	Not at all	N/A *
Can you clearly determine when the child is happy?	**45%**	40%	12.5%	0%	2.5%	0%
Can you recognize when the child is in pain?	10%	30%	**47.5%**	10%	2.5%	0%
Can you assess the intensity of the pain the child is experiencing?	5%	7.5%	25%	25%	**37.5%**	0%
Can you recognize when the child is ill?	17.5%	30%	**47.5%**	5%	0%	0%
Can you recognize when the child is anxious/nervous?	25%	**40%**	27.5%	2.5%	5%	0%
Can you identify what causes the child’s anxiety?	0%	27.5%	**32.5%**	17.5%	20%	2.5%
Can you anticipate the emergence of inappropriate behaviors in the child, such as aggression?	7.5%	**35%**	**35%**	5%	15%	2.5%
Do you know if the child has hyposensitivity?	17.5%	17.5%	25%	5%	30%	5%
Do you know if the child has hypersensitivity?	10%	25%	12.5%	15%	32.5%	5%
For children who have used non-technological means to communicate their emotions, was it helpful for them?	9.09%	**45.45%**	18.18%	18.18%	9.09%	0%

* N/A: Not Available/Unknown.

## Data Availability

The original contributions presented in this study are included in the article. Further inquiries can be directed to the corresponding author.

## Abbreviations

The following abbreviations are used in this manuscript:

ASD	Autism Spectrum Disorder
nmvASD	Non or Minimally Verbal Autism
ABA	Applied Behavior Analysis
PECS	Picture Exchange Communication System
EIBI	Early Intensive Behavioral Intervention
DSM-5	Diagnostic and Statistical Manual of Mental Disorders
HR	Heart Rate
HRV	Heart Rate Variation
GSR	Galvanic Skin Response
EDA	Electrodermal Activity
EEG	Electroencephalogram
ECG	Electrocardiogram

## Appendix A. Inclusion Criteria

Inclusion Criteria
ASD diagnosed by an expert in child psychiatry Aged 3 years to 17 years 11 months
Severe language impairment: non or minimally verbal
